# Neurological outcomes and duration from cardiac arrest to the initiation of extracorporeal membrane oxygenation in patients with out-of-hospital cardiac arrest: a retrospective study

**DOI:** 10.1186/s13049-017-0440-7

**Published:** 2017-09-16

**Authors:** Takahiro Yukawa, Masahiro Kashiura, Kazuhiro Sugiyama, Takahiro Tanabe, Yuichi Hamabe

**Affiliations:** Trauma and Critical Care Center, Tokyo Metropolitan Bokutoh Hospital, 23-15 Kohtohbashi, 4-Chome, Sumida-ku, Tokyo, 130-8575 Japan

**Keywords:** Cardiac arrest, Extracorporeal cardiopulmonary resuscitation, Extracorporeal membrane oxygenation, Neurological outcome

## Abstract

**Background:**

We investigated the relationship between neurological outcomes and duration from cardiac arrest (CA) to the initiation of extracorporeal membrane oxygenation (ECMO) (CA-to-ECMO) in patients with out-of-hospital cardiac arrest (OHCA) treated with extracorporeal cardiopulmonary resuscitation (ECPR) and determined the ideal time at which ECPR should be performed.

**Methods:**

During the time period in which this study was conducted, 3451 patients experienced OHCA. This study finally included 79 patients aged 18 years or older whose OHCA had been witnessed and who underwent ECPR in the emergency room between January 2011 and December 2015. Our primary endpoint was survival to hospital discharge with good neurological outcomes (a cerebral performance category of 1 or 2).

**Results:**

Of the 79 patients included, 11 had good neurological outcomes. The median duration from CA-to-ECMO was significantly shorter in the good neurological outcome group (33 min, interquartile range [IQR], 27–50 vs. 46 min, IQR, 42–56: *p* = 0.03). After controlling for potential confounders, we found that the adjusted odds ratio of CA-to-ECMO time for a good neurological outcome was 0.92 (95% confidence interval: 0.87–0.98, *p* = 0.007). The area under the receiver operating characteristic curve of CA-to-ECMO for predicting a good neurological outcome was 0.71, and the optimal CA-to-ECMO cutoff time was 40 min. The dynamic probability of survival with good neurological outcomes based on CA-to-ECMO time showed that the survival rate with good neurological outcome decreased abruptly from over 30% to approximately 15% when the CA-to-ECMO time exceeded 40 min.

**Discussion:**

In this study, CA-to-ECMO time was significantly shorter among patients with good neurological outcomes, and significantly associated with good neurological outcomes at hospital discharge. In addition, the probability of survival with good neurological outcome decreased when the CA-to-ECMO time exceeded 40 minutes. The indication for ECPR for patients with OHCA should include several factors. However, the duration of CPR before the initiation of ECMO is a key factor and an independent factor for good neurological outcomes in patients with OHCA treated with ECPR. Therefore, the upper limit of CA-to-ECMO time should be inevitably included in the indication for ECPR for patients with OHCA. In the present study, there was a large difference in the rate of survival to hospital discharge with good neurological outcome between the patients with a CA-to-ECMO time within 40 minutes and those whose time was over 40 minutes. Based on the present study, the time limit of the duration of CPR before the initiation of ECMO might be around 40 minutes. We should consider ECPR in patients with OHCA if they are relatively young, have a witness and no terminal disease, and the initiation of ECMO is presumed to be within this time period.

**Conclusions:**

The duration from CA-to-ECMO was significantly associated with good neurological outcomes. The indication for patients with OHCA should include a criterion for the ideal time to initiate ECPR.

## Background

Extracorporeal cardiopulmonary resuscitation (ECPR) is a novel CPR technique that uses veno-arterial extracorporeal membrane oxygenation (ECMO). Some observational studies have shown that, compared to conventional CPR, ECPR has a beneficial effect on survival and neurological outcomes in patients with both in-hospital and out-of-hospital cardiac arrest (CA) [1–4]. Based on the 2015 American Heart Association guideline, ECPR is given a class IIb recommendation if it is performed rapidly in a specialized center, and should be used in patients with a potentially reversible etiology. Thus, the use of ECPR is increasing rapidly in emergency departments for patients with CA as an alternative for resuscitation.

However, the definite indication for ECPR has not been established, especially in patients with out-of-hospital cardiac arrest (OHCA) [5]. It is obvious that prolonged cardiac arrest without the return of spontaneous circulation (ROSC) precludes the possibility of ECPR. However, the acceptable duration from CA to the initiation of ECMO to achieve good neurological outcomes has not been clarified.

We sought to investigate the relationship between neurological outcomes and duration from CA to the initiation of ECMO in patients with OHCA treated with ECPR, and to determine the ideal time at which ECPR should be performed in these patients.

## Methods

### Setting and participants

In this retrospective study, we included witnessed patients with OHCA aged 18 years or older who were brought to the emergency department of the Tokyo Metropolitan Bokutoh Hospital in Japan and were treated with ECPR between January 2011 and December 2015. In Japan, emergency medical personnel are allowed to perform defibrillation, tracheal intubation, and injection of adrenaline (epinephrine), but they are not allowed to administer anti-arrhythmic drugs such as amiodarone. Chest compression is performed manually in most cases. The institutional review board of the Tokyo Metropolitan Bokutoh Hospital approved the performance of this study (approval number: 61, 2016).

### ECPR protocol, technique, and devices

The indications for ECPR in our institution are as follows: (i) patients aged ≤65 years whose OHCA was witnessed by a bystander and in whom an initial shockable rhythm was found; or (ii) patients aged ≤70 years whose OHCA was witnessed by emergency medical service (EMS) personnel and who were presumed to have a reversible underlying etiology, regardless of the initial rhythm. ECPR is not performed if it takes a very long time for the patient to be transferred to hospital (e.g., 60 min or more). The implementation of ECPR is finally decided at the discretion of the individual emergency physician; therefore, some cases did not meet the rigid indications for ECPR.

ECPR was implemented immediately after the patient’s arrival at the emergency room. In all cases, we selected the ipsilateral or contralateral femoral vein and artery for cannulation of venous and arterial cannulas. Cannulas of sizes 15–16 French (Fr) were chosen for the femoral artery, and sizes 21–22 Fr were chosen for the femoral vein. Cannulation was performed percutaneously with the Seldinger technique under an ultrasonic guide.

Our emergency room was renovated, and interventional radiology-computed tomography was introduced in the emergency room in August 2014. Until July 2014, a cannula was placed under ultrasonic guidance only, but under both ultrasonic and fluoroscopic guidance after August 2014.

The ECMO circuit, including centrifugal pump, hollow-fiber oxygenator (MERA CPB circuit: Senko Medical Instrument Mfg. Corp., Tokyo, Japan or Capiox EBS; Terumo Corp., Tokyo, Japan) and a heparin-coated surface circuit, was primed using normal saline with 3000 units of heparin.

After venous and arterial cannulas were successfully inserted, the ECMO circuit was connected and the ECMO pump flow was set between 3 and 4 L/min at the start. After the start of ECMO, a 4-Fr sheath was placed in the superficial femoral artery to prevent distal limb ischemia.

### Post cardiac arrest care

After the ECMO pump was turned on, we immediately examined the electrocardiogram and assessed whether the current coronary disease was related. Emergent coronary angiography was performed for patients who were presumed to suffer from acute coronary syndrome (ACS). Uninterrupted percutaneous coronary intervention was performed if necessary.

After admission to the intensive care unit, the patients were managed with post-resuscitation care [6] by: (i) maintaining the mean arterial pressure at more than 65 mmHg; (ii) managing the target body temperature at 34 °C until 24 h from starting the ECMO pump, then warming it up to 36 °C at the rate of 0.15 °C per h with a heat exchanger; (iii) decreasing cardiac afterload as much as possible; (iv) properly titrating FiO_2_ and positive end-expiratory pressure at the respirator.

### Data collection, study definitions, and endpoints

We extracted data from the patients’ electronic medical records retrospectively. The timing of pre-hospital events was recorded according to the reports of emergency medical service personnel. The onset of CA was estimated from the report of the witness. If this information was not available, the timing of the emergency call was regarded as the onset of CA.

We collected data on age, sex, initial rhythm, existence of bystander CPR, existence of transient ROSC before hospital arrival, time from CA to arrival at the hospital (CA-to-arrival time), time from arrival at the hospital to the initiation of ECMO (arrival-to-ECMO time), and total time from CA to the initiation of ECMO (CA-to-ECMO time).

The primary endpoint was survival to hospital discharge with good neurological outcomes. The cerebral performance categories (CPC) scale was used to assess neurological outcomes at hospital discharge. The neurological outcome was considered good if the CPC score was 1 or 2 and poor if the CPC score was 3–5 [7].

### Statistical analyses

Descriptive statistics were calculated for all variables of interest. Continuous variables were reported as medians and interquartile ranges (IQRs), and categorical variables were summarized using counts and percentages. Univariate analysis was performed using Fisher’s exact test for categorical variables and the Mann-Whitney U test for continuous variables. All reported *p*-values were two-tailed, and values less than 0.05 were considered statistically significant. All statistical analyses were performed using EZR (Saitama Medical Center, Jichi Medical University, Saitama, Japan), which is a graphical user interface for R (The R Foundation for Statistical Computing, Vienna, Austria) [8].

CA-to-ECMO time was compared between the good and poor neurological groups. A multivariate logistic regression analysis was then performed to evaluate the association between CA-to-ECMO time and good neurological outcomes to control for potential confounders. We selected potential confounders that appeared to be clinically important by referring to those used in previous studies (age, sex, initial shockable rhythm, and existence of transient ROSC before hospital arrival) [4, 9, 10]. The optimal CA-to-ECMO time cutoff for predicting a good neurological outcome was determined using the Youden index. Additionally, we calculated the dynamic probability of survival to hospital discharge with good neurological outcomes by CA-to-ECMO time.

## Results

**Fig. 1:**
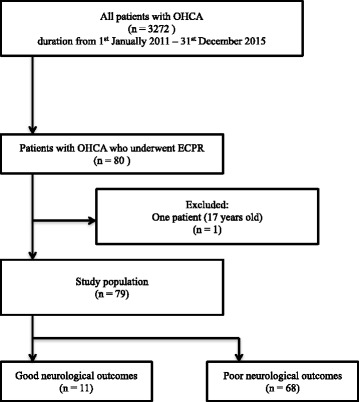
Selection of study patients. OHCA: out-of-hospital cardiac arrest, ECPR: extracorporeal cardiopulmonary resuscitation

During the time period in which this study was conducted, 3451 patients experienced OHCA. Of these, 80 patients were treated with ECPR at our institution. One patient was excluded because he was 17 years old; therefore, 79 patients were finally included in the study (Fig. 1).

Table 1 shows the baseline characteristics and post-CA care of all patients and both the good and poor neurological outcome groups. The median age of the patients was 59 years (IQR, 48–64); 66 patients (83%) were men. Fifty-nine patients had an initial shockable rhythm. Twenty-one patients (27%) recovered spontaneous circulation transiently prior to admission to the hospital, but went into cardiac arrest again before arrival at hospital. Seventeen patients (22%) survived until hospital discharge. Of these, 11 cases (14%) had good neurological outcomes (CPC score, 1 or 2). Sixty-two patients (79%) died in the hospital; therefore, 68 patients (86%) had poor neurological outcomes (CPC score, 3–5). The proportion of patients whose OHCA was witnessed by EMS personnel was significantly higher among patients with good neurological outcomes than those with poor outcomes (73% vs. 19%; *p* < 0.01). Additionally, the proportion of patients with bystander CPR was significantly higher among patients with good neurological outcomes (91% vs. 53%; *p* = 0.02).

**Table 1:** Comparison of baseline characteristics and post-CA care between the good and poor neurological outcome groups

	All [median (IQR)] *n* = 79	Good neurological outcomes [median (IQR)] *n* = 11	Poor neurological outcomes [median(IQR)] *n* = 68	*P*-value
Age	59.0 [48.5–64.5]	61.0 [42.5–67.5]	58.5 [51.3–64.0]	0.854
Sex (Male)	65 (82.3%)	6 (54.5%)	59 (86.8%)	0.021
Initial shockable rhythm	58 (73.4%)	6 (54.5%)	52 (76.5%)	0.150
EMS personnel witness	21 (26.6%)	8 (72.7%)	13 (19.1%)	0.001
Bystander CPR	46 (58.2%)	10 (90.9%)	36 (52.9%)	0.021
Existence of transient ROSC before hospital arrival	21 (26.6%)	6 (54.5%)	15 (22.1%)	0.059
Pre-hospital epinephrine use	18 (22.8%)	2 (18.2%)	16 (23.5%)	1
Pre-hospital defibrillation	58 (73.4%)	7 (63.6%)	51 (75.0%)	0.470
Cause of CA (ACS)	39 (48.8%)	5 (45.5%)	34 (50.0%)	1
Initial creatinine	1.10 (1.00–1.30)	1.10 [0.90–1.45]	1.10 [1.00–1.30]	0.854
Initial lactate	14.0 (10.8–16.0)	16.0 [10.7–16.5]	13.6 [10.8–16.0]	0.509
CAG performed	50 (62.5%)	7 (63.6%)	43 (63.2%)	1
PCI performed	26 (32.5%)	4 (36.4%)	22 (32.4%)	1
Induction of TTM	50 (62.5%)	9 (81.8%)	41 (60.3%)	0.312
Successful weaning from ECMO	24 (30.0%)	11 (100%)	13 (19.1%)	<0.001
Length of ICU stay	3.0 (1.0–11.0)	14.0 (11.5–19.5)	2.5 [1.0–7.3]	<0.001

*IQR* interquartile range, *EMS* emergency medical service, *CPR* cardiopulmonary resuscitation, *ROSC* return of spontaneous circulation, *CA* cardiac arrest, *ACS* acute coronary syndrome, *CAG* coronary angiography, *PCI* percutaneous coronary intervention, *TTM* targeted temperature management; ECMO, extracorporeal membrane oxygenation

The median CA-to-arrival time was significantly shorter among patients with good neurological outcomes than those with poor outcomes (17 min [IQR, 3–25] vs. 28 min [IQR, 22–33]; *p* = 0.03). The median arrival-to-ECMO time was comparable between the groups (19 min [IQR, 14–31] vs. 20 min [IQR, 14–25]; *p* = 0.03). The median collapse-to-ECMO time was significantly shorter among patients with good neurological outcomes (33 min [IQR, 27–50] vs. 46 min [IQR, 42–56]; *p* = 0.03) (Table 2).

**Table 2:** Comparison of the duration from cardiac arrest to hospital arrival (CA-to-arrival), hospital arrival to the initiation of extracorporeal membranous oxygenation (arrival-to-ECMO) and cardiac arrest to the initiation of extracorporeal membranous oxygenation (CA-to-ECMO) between the good and poor neurological outcome groups

	All [median (IQR)] *n* = 79	Good neurological outcomes [median (IQR)] *n* = 11	Poor neurological outcomes [median (IQR)] *n* = 68	*P*-value
CA-to-arrival	27.0 [18.5–32.5]	17.0 [2.5–24.5]	28.0 [21.8–33.3]	0.007
arrival-to-ECMO	19.0 [14.0–26.0]	19.0 [14.0–30.5]	19.5 [14.0–25.3]	0.723
CA-to-ECMO	45.0 [40.0–56.5]	33.0 [26.5–49.5]	45.5 [41.8–56.3]	0.026

*IQR* interquartile range, *CA* cardiac arrest, *ECMO* extracorporeal membrane oxygenation

There are twenty-one patients who did not have an initial shockable rhythm, and thus qualified for exclusion based on our criteria for ECPR. This group was limited to patients with good neurological outcomes, and their diseases tended to be specific such as acute pulmonary embolism, and hypoxia due to acute respiratory failure (acute heart failure, bronchiolar asthma attack, and others). These patients were given epinephrine intravenously by EMS personnel, and consequently had ROSC at least once.

After controlling for potential confounders, we found that the adjusted odds ratio of CA-to-ECMO time for good neurological outcomes was 0.92 (95% confidence interval: 0.87–0.98, *p* < 0.01). In this model, male sex and transient ROSC before hospital arrival were also significantly associated with good neurological outcomes (Table 3). The adjusted odds ratio of CA-to-arrival adjusted by the same confounders was 0.91 (95% confidence interval; 0.85–0.97, *p* < 0.01).

**Table 3:** Multivariate logistic regression analysis of prognostic factors of good neurological outcome (CPC 1 or 2) at hospital discharge

	Adjusted odds ratio	[95% CI]	*P*-value
Age	0.996	[0.93–1.06]	0.902
Male gender	0.098	[0.013–0.73]	0.023
Initial shockable rhythm	2.030	[0.26–15.90]	0.499
Existence of transient ROSC before hospital	8.170	[1.28–52.20]	0.027
CA-to-ECMO time (per minute)	0.907	[0.85–0.97]	0.007

*CPC* cerebral performance category, *ROSC* return of spontaneous circulation, *CA* cardiac arrest, *ECMO* extracorporeal membrane oxygenation

**Fig. 2:**
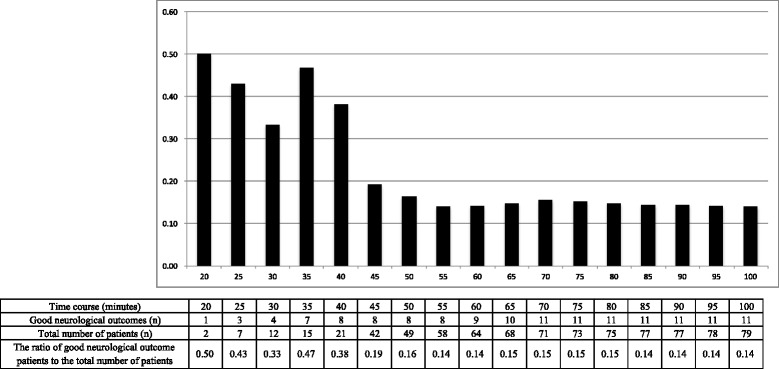
The dynamic probability of survival to hospital discharge with good neurological outcome based on the time from witnessed CA to the initiation of ECMO. The survival rate with good neurological outcome was 38% when the duration of CPR was within 40 min; however, it decreased to about 15% when CPR was performed for more than 40 min. CA: cardiac arrest, ECMO: extracorporeal membrane oxygenation, CPR: cardiopulmonary resuscitation

Figure 2 shows the dynamic probability of survival to hospital discharge with good neurological outcome by CA-to-ECMO time. The rate of survival decreased abruptly when the CA-to-ECMO time exceeded 40 min wherein the rate of survival reduced to approximately 14%. Drawing a receiver operating characteristic curve of CA-to-ECMO time for good neurological outcome patients, we found that the optimal cutoff was 40.00 (sensitivity: 0.73, specificity 0.81), and the area under the curve was 0.71.

Three patients had a good neurological outcome at hospital discharge, although their CA-to-ECMO time was longer than 40 min. These three patients had common characteristics: they had transient ROSC before hospital arrival and an initial shockable rhythm.

## Discussion

In this study, CA-to-ECMO time was significantly shorter among patients with good neurological outcomes, and significantly associated with good neurological outcomes at hospital discharge. In addition, the probability of survival with good neurological outcome decreased when the CA-to-ECMO time exceeded 40 min.

Several studies have shown that ECPR in OHCA patients may be a good alternative to conventional CPR [3, 4, 6]. The reported survival rate with minimal disability ranged widely from 5%–30% [3, 4, 11–14]. However, the human and economic cost of ECMO management cannot be negligible. ECPR for patients with OHCA could reduce the severe burden on the hospital’s emergency department; however, to our knowledge, no study has investigated the cost effectiveness of ECPR for patients with OHCA. Adequate patient selection with strict indication criteria is essential if ECPR becomes a part of the standard care of patients with OHCA.

The indication for ECPR for patients with OHCA should include several factors including age, the presence of witnesses, bystander CPR, previous medical status, arterial pH, and serum lactate concentration at hospital admission, shockable cardiac rhythm, and transitory ROSC [10, 15]. However, the duration of CPR before the initiation of ECMO is a key factor for good neurological outcomes in patients with OHCA treated with ECPR. Moreover, in our study, the CA-to-ECMO time was shown to be an independent prognostic factor for good neurological outcomes. Therefore, the upper limit of CA-to-ECMO time should be inevitably included in the indication for ECPR for patients with OHCA. In inpatients with -hospital cardiac arrest (IHCA), Chen et al. reported that prolonged resuscitation more than 60 min before the initiation of ECMO was associated with poor neurological outcomes [16]. The prognosis of patients with IHCA treated with ECPR tended to be better than that of patients with OHCA [11, 17]. This is due to situational differences between patients with IHCA and those with OHCA. In patients with IHCA, CPR is more likely to be started immediately; furthermore, the quality of CPR and advanced life support is more secure, and the duration before the achievement of ECMO flow is shorter. Therefore, the ideal time criteria for ECPR should be different between patients with OHCA and those with IHCA. However, studies that discuss the time criteria in patients with OHCA are scarce. Hase et al. reported that the optimal cutoff duration of CPR for a good outcome was 45 min in patients with OHCA treated with ECPR [18]. In the present study, there was a large difference in the rate of survival to hospital discharge with good neurological outcome between the patients with a CA-to-ECMO time within 40 min and those whose time was over 40 min. The survival rate with good neurological outcome was about 35% when the duration of CPR was within 40 min; however, it decreased to about 15% if CPR was performed for over 40 min. This is similar to the result of the study by Hase et al. The acceptable survival rate to make ECPR in patients with OHCA cost effective is not clear. However, we think it is reasonable to regard the point at which there is a large difference in the survival rate as the ideal time. Based on the present study, the time limit of the duration of CPR before the initiation of ECMO might be around 40 min. We should consider ECPR in patients with OHCA if they are relatively young, have a witness and no terminal disease, and the initiation of ECMO is presumed to be within this time period. In other words, we should make every effort in both pre-hospital and in-hospital care to establish ECMO within 40 min from the onset of cardiac arrest. In our study, arrival-to-ECMO time was a median of 19 min, and about 10 min in faster cases. The acceptable duration prior to hospital admission was therefore limited and at most 30 min.

However, it is also true that time alone is not an absolute criterion, and some patients have good neurological outcome regardless of prolonged CPR. It is very important to find prognostic factors that predict good neurological outcomes in patients with prolonged CPR. In our study, three patients had good neurological outcomes, although their CA-to-ECMO time exceeded 40 min, and they all recovered spontaneous circulation transiently before hospital arrival. In these patients, cerebral perfusion could be slightly maintained around the duration of ROSC, and a longer time might be permissible. Recently, some physiological parameters specific to the individual patient during CPR, such as regional saturation of oxygen in the brain, have been reported to be able to predict neurological outcome in patients with OHCA [19, 20]. In the future, such parameters could be used to indicate patients with the potential for neurological recovery, regardless of prolonged CPR duration.

## Limitations

There are several limitations to this study. First, it was a retrospective study with a small sample size. Second, this study lacks some information about post-cardiac arrest care, such as transfusion, ventilator setting, and therapies for infectious complications. Additionally, explanatory variables in the logistic regression analysis were limited because of the small sample size. Therefore, some potential prognostic factors could not be adjusted. Third, neurological outcomes were evaluated only at the time of hospital discharge. CPC scores and/or survival rate after hospital discharge at 3 months or 6 months, for example, were not evaluated. Finally, the outcome of ECPR for patients with OHCA is affected by the system of emergent medical services and pre-hospital treatment. It is well known than manual CPR is difficult to perform in a moving ambulance, and mechanical CPR in this situation could provide different results with a varying optimal CPR duration before ECPR initiation. Therefore, the relationship between the prognosis and the duration of CPR before the initiation of ECMO might be different in other settings than ours. A well-designed, prospective, multicenter study with a large sample size is needed to determine the standard indication for ECPR for patients with OHCA.

## Conclusion

The survival rate with good neurological outcome decreased when the duration until the initiation of ECMO exceeded 40 min in patients with OHCA treated with ECPR. The indication for ECPR in patients with OHCA should include a time criterion. Based on the present study, this ideal time might be within 40 min from the onset of CA.
